# Catching Fire: *Candida albicans*, Macrophages, and Pyroptosis

**DOI:** 10.1371/journal.ppat.1004139

**Published:** 2014-06-26

**Authors:** Damian J. Krysan, Fayyaz S. Sutterwala, Melanie Wellington

**Affiliations:** 1 Department of Pediatrics, University of Rochester School of Medicine and Dentistry, Rochester, New York, United States of America; 2 Department of Microbiology and Immunology, University of Rochester School of Medicine and Dentistry, Rochester, New York, United States of America; 3 Inflammation Program, University of Iowa Carver College of Medicine, Iowa City, Iowa, United States of America; 4 Department of Internal Medicine, University of Iowa Carver College of Medicine, Iowa City, Iowa, United States of America; 5 Veterans Affairs Medical Center, Iowa City, Iowa, United States of America; The University of North Carolina at Chapel Hill, United States of America

## Introduction

*Candida albicans* is a commensal organism of the human gastrointestinal and genitourinary systems as well as the most common human fungal pathogen [1]. The organism causes mucosal infections such as oropharyngeal or vulvo-vaginal candidiasis, but it can also cause life-threatening invasive disease. Healthy individuals readily maintain the organism in its commensal state but individuals with defects in the anti–*C. albicans* immune response are at high risk for developing disease. Phagocytes, particularly macrophages and neutrophils, are critical to the host's ability to prevent invasive candidiasis [2].

*C. albicans* poses a particular challenge to host defenses because it is polymorphic; round yeast forms and filamentous pseudohyphae and hyphae forms are all present during infection [3], [4]. Yeast and filamentous *C. albicans* have a number of important physiological, structural, and biochemical differences. Accordingly, the immune response to these forms differs substantially [4], [5]. The type of recognition receptors used to detect *C. albicans*, as well as the phagocyte type, anatomic site of infection, and course of infection, all serve to modulate the host response to the organism [2], [6]. Macrophages are particularly important because they can both limit *C. albicans* burden early in infection and recruit and activate other immune effector cells [4], [6]. Our understanding of the mechanisms and consequences of the interaction between macrophages and *C. albicans* is improving, but much remains to be learned.

## What Does the Macrophage–*C. albicans* Interaction “Look” Like?

Much of our understanding of the interaction between *C. albicans* and macrophages arose from observations using wide-field, confocal, and fluorescence microscopy. Macrophages readily ingest the round yeast form of *C. albicans* as well as relatively short *C. albicans* filaments [7]. After ingestion, some *C. albicans* are killed; however, most survive and form hyphae in response to the phagosome environment (morphogenesis) [8]. Time-lapse microscopy suggests that some macrophages are able to withstand the stress of elongating *C. albicans* filaments without apparent loss of integrity, whereas other macrophages that have ingested *C. albicans* undergo lysis [9]. As lysis is temporally linked to filament elongation, the filaments appear to puncture through macrophage membrane [2], [9], [10]. Thus, while macrophages are able to damage or kill *C. albicans*, the fungus also has a significant cytotoxic effect on macrophages.

In addition to their role in ingestion and possible clearance of *C. albicans*, macrophages make a critical contribution to the innate and adaptive anti–*C. albicans* immune response. Macrophages produce a variety of pro- and anti-inflammatory cytokines in response to *C. albicans*; the type of response is governed both by morphology and other organisms factors as well as by the host pathogen recognition receptors (PRR) that are engaged (see *PLOS Pathogens* Pearls [11] and [3] or, for a more in-depth review, [4]). In particular, hyphae formation is a strong trigger for production of the pro-inflammatory cytokine interleukin-1β (IL-1β) [5], [12].

## How Do *C. albicans* Filaments Kill Macrophages?

Because of the visual/temporal association of intracellular filament growth with macrophage lysis, a logically appealing hypothesis is that *C. albicans* filaments simply grow so long that the macrophage membrane is stretched to the point of failure, resulting in lysis [2], [5]. Two additional findings support this hypothesis: First, killed or inactivated *C. albicans* yeast, which obviously do not form filaments within macrophages, trigger minimal levels of macrophage lysis [13]. Second, *C. albicans* mutant strains that do not form filaments also do not trigger macrophage lysis [10].

Despite the appealing simplicity of the physical rupture hypothesis, conflicting data has emerged. Several laboratories have identified *C. albicans* mutant strains that form normal hyphae within macrophages yet induce significantly lower levels of lysis [10], [14], [15]. Furthermore, recent time lapse microscopy experiments have observed “non-lytic expulsion/exocytosis,” in which *C. albicans* hyphae appear to be expelled from within macrophages without loss of macrophage viability [16]. Thus, hyphal formation alone is not sufficient to trigger macrophage lysis.

## Are Macrophage Programmed Cell Death Pathways Activated by *C. albicans*?

An alternative to the long-held idea that *C. albicans* physically destroys macrophages is that macrophage lysis in response to *C. albicans* is actually a macrophage-driven response. In the last decade, there has been an explosion of new data describing programmed cell death pathways in response to infection [17]. The designation “programmed” refers to cell death that is specifically induced by host-cell signaling pathways; thus, programmed cell death is host-driven. The archetypal programmed cell death pathway apoptosis may occur in macrophages responding to *Candida* [18]; however, apoptosis is non-lytic and cannot account for *C. albicans*–induced lysis. In contrast, several newly described programmed cell death pathways result in lytic cell death, including: pyroptosis, pyronecrosis, and necroptosis [17]. The most well studied of these is pyroptosis, which results in cell swelling, lysis, and release of inflammatory cytokines (see the *PLOS Pathogens* Pearl [19], or [20] for more detail). This pathway was originally identified in macrophages infected with intracellular bacteria such as *Salmonella*, *Legionella*, and possibly *Mycobacteria*. By undergoing pyroptosis, infected macrophages deprive intracellular bacteria of their immune-protected niche as well as intracellular nutrients.

A critical hallmark of pyroptosis is its dependence on the cysteine protease caspase-1 [17]. Caspase-1 is activated via formation of the inflammasome, a multiprotein complex that forms in response to a variety of inflammatory signals. Inflammasome formation is initiated through activation of either a nod-like receptor protein (NLRP1, NLRP3, or NLRC4) or the absent in melanoma protein AIM2 [21]. Subsequently, the adaptor molecule ASC (apoptosis-associated speck-like protein containing a CARD) is recruited to the complex; this is followed by binding and activation of caspase-1. In addition to its role in triggering cell lysis, activation of caspase-1 results in cleavage of the pro-forms of the cytokines IL-1β and IL-18 into their mature forms [20]. Thus, pyroptosis is a lytic, inflammatory form of cell death. When considering the role of caspase-1 in pyroptosis, it should be noted that the caspase-1 deficient mice used in the majority of research studies are not only deficient for caspase-1 but also have a dysfunctional caspase-11 [22].

**Figure 1:**
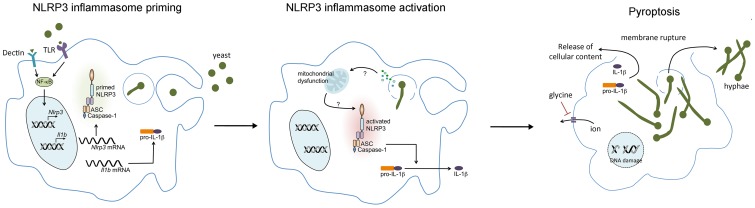
*C. albicans*–mediated NLRP3 inflammasome activation. Upon encountering *C. albicans*, pattern recognition receptors (PRR) on the macrophage, such as TLR2 and Dectin 1 and 2, activate NF-κB, leading to the transcription and translation of NLRP3 and pro-IL-1β. Phagocytosis of *C. albicans* yeast forms triggers hyphal formation which may result in lysosomal rupture. NLRP3 inflammasome activation is then triggered through an as-yet-undefined mechanism. Although morphogenesis appears to be necessary for inflammasome activation, it is not sufficient. Activation of the NLRP3 inflammasome results in activation of the cysteine protease caspase-1, which mediates the processing and secretion of pro-IL-1β and pro-IL-18. Caspase-1 activation also induces pyroptotic cell death of the macrophage, resulting in cell swelling, DNA fragmentation, and the lytic release of intracellular inflammatory contents. Osmotic lysis of the macrophage during pyroptosis can be inhibited by the addition of extracellular glycine.

*C. albicans* triggers the activation of NLRP3, NLRC4, and noncanonical inflammasomes [23]–[27], suggesting that *C. albicans* might trigger macrophage lysis via pyroptosis. Studies from our laboratory and from Uwamahoro et al., recently demonstrated that the majority of *C. albicans*–induced macrophage lysis requires caspase-1 [13], [15]. We also found that NLRP3 and ASC, but not NLRC4, were required for this process [13]. Thus, host cell components are required for *C. albicans*–induced lysis; this would not be expected if lysis were due to physical disruption of the macrophage by *C. albicans* filaments. Furthermore, *C. albicans*-induced macrophage lysis can be substantially suppressed by the addition of glycine to the culture medium [13]. Glycine, which has no effect on *C. albicans* growth or filamentation, suppresses pyroptotic lysis, presumably via blocking membrane pores [28]. Taken together, these data clearly demonstrate that pyroptosis occurs in response to *C. albicans* via the NLRP3 inflammasome (Figure 1). Thus, most of the “cytotoxicity” seen in macrophages exposed to *C. albicans* is controlled not by *C. albicans* but rather by host cell pathways [13]. The role of other lytic programmed cell death pathways, such as pyronecrosis or necroptosis, has not been studied, and it remains possible that these pathways are also triggered by *C. albicans*. Nevertheless, pyroptosis appears to play a major role in the lytic response of macrophages to ingested *C. albicans* cells.

Our results with mutant *C. albicans* strains demonstrated that pyroptotic lysis in response to *C. albicans* does not require hyphal formation [14]. However, we have also observed that the non-*albicans Candida* species and *Saccharomyces cerevisiae* strains that are capable of forming filaments are stronger inducers of pyroptosis than those that do not form filaments [13]. Thus, morphogenesis appears to be necessary but not sufficient for triggering pyroptosis. We expect to be able to use this set of *Candida* and *Saccharomyces* strains and mutants as a powerful tool for future investigations into the mechanisms through which *C. albicans* triggers pyroptosis.

## How Does Inflammasome Activation Trigger Inflammation in Response to *C. albicans*?

Another important consequence of pyroptosis is the release of IL-1β/IL-18 [20]. The finding that pyroptosis occurs in response to *C. albicans* is quite consistent with the ability of *C. albicans* to trigger IL-1β production in macrophages. Production of mature IL-1β via the NLRP3 inflammasome is tightly regulated in a two-step process: The first, or priming signal, triggers activation of NFκB and transcription of pro-IL-1β [12]. The second signal results in inflammasome assembly, caspase-1 activation, and cleavage of pro-IL-1β into mature IL-1β. As with most biological systems, the two signal “pathways” are not completely separated; priming signals also increase the level of NLRP3 [29].

Macrophages have a variety of PRR that recognize *C. albicans* and may provide the first or priming signal for inflammasome activation. These include complement receptors; the C-type lectins dectin-1, dectin-2 and mannose receptor; and Toll-like receptors, particularly TLR2 [3], [4], [11]. The mechanism(s) by which *C. albicans* provides the second signal for inflammasome activation is less clear. The NLRP3 inflammasome is activated in response to a wide range of stimuli including cellular stress, tissue damage, and many types of infection [29]. It appears that NLRP3 responds to these myriad conditions through their convergence on mitochondrial damage; potassium efflux, calcium influx and increased mitochondrial reactive oxygen species production are common triggers of activation. In addition, lysosomal rupture is required for NLRP3 inflammasome activation in response to particulate agonists. Any combination of these signals may occur in *C. albicans* exposed macrophages; furthermore, the mechanisms of *C. albicans* mediated NLRP3 activation may vary in different environmental conditions and phagocyte types. As with *C. albicans*–induced macrophage lysis, activation of NLRP3 was thought to occur in direct response to hyphal formation [5]. However, as with our studies on pyroptosis, our data suggests that, at least for macrophages, morphogenesis is necessary but not sufficient to trigger NLRP3 activation [14].

In addition to the NLRP3 inflammasome, C. *albicans* activates the NLRC4 inflammasome as well as a noncanonical caspase-8 containing inflammasome [26], [27]. Some of the variation in inflammasome responses to *C. albicans* may be related to the type of host cell that encounters the organism, the PRR used, and/or the type of infection. This is exemplified by the finding that NLRC4 expressed in mucosal stromal cells is important in defense against oropharyngeal candidiasis [26]. In addition, activation of the 1,3-β-glucan lectin dectin-1 on dendritic cells leads to production of IL-1β via a noncanonical inflammasome that utilizes caspase-8 rather than caspase-1 [27]. The role of caspase-8 in production of IL-1β raises particularly interesting questions about caspase activation and programmed cell death pathways as caspase-8 also has a prominent role in initiating apoptosis. Clearly, there is still much to learn about the role of cell death pathways, inflammasome activation, and cytokine production in *C. albicans* infections.

IL-1β production in response to *C. albicans* is important for recruiting additional phagocytes to the site of infection and stimulating protective immune responses via the T_h_17 and/or T_h_1 pathway [30]. Less is known about the role of IL-18 in the host response to *C. albicans*, but it has been associated with recruitment of monocytes to the site of infection, the development of protective T_h_1 responses, and modest increases in the ability of neutrophils to damage *C. albicans* pseudohyphae [30], [31].

Pyroptosis also triggers inflammation through IL-1β/IL-18 independent pathways, including production of IL-1α, HMGB1, and eicosanoids [19]; these danger signals may be important in anti–*C. albicans* defenses. Inflammasome activation in response to *C. albicans* has been implicated in elaboration of IL-6, CXCL1 (a murine chemokine similar to human IL-8), and antimicrobial peptides [26]. Furthermore, cell lysis results in global release of intracellular molecules such as ATP, DNA, RNA, which are inflammatory when found in the extracellular environment [19]. Very little is known about the role that these molecules and processes play in the inflammatory response to *C. albicans*, but they may represent additional mechanisms through which pyroptosis contributes to the anti–*C. albicans* host defense.

## Summary: A New Paradigm for Host–*C. albicans* Interactions

Although *C. albicans* hyphae formation clearly plays a role in macrophage lysis, the death of macrophages that have ingested *C. albicans* is not simply the result of the hyphae physically rupturing the macrophage [13]. Rather, the current data supports a new model in which *C. albicans*–induced macrophage lysis occurs via pyroptosis, a host-cell programmed death pathway. These findings represent the first demonstration that pyroptosis occurs in response to a fungal pathogen. One important question raised by these findings is whether pyroptosis is beneficial to the host, *C. albicans*, or both. The components of the NLRP3 inflammasome as well as IL-1β, IL-18, and the IL-1α/IL-1β receptor IL-1RI, are important for host survival from systemic candidiasis and prevention of dissemination of oropharyngeal candidiasis [12], [32]. Thus, the NLRP3 inflammasome is clearly important to the host for its role in triggering inflammation; it may also benefit the host by triggering pyroptosis. Alternatively, the host program of pyroptosis could have been “conscripted” during the evolution of *C. albicans* to provide a mechanism of escape from the macrophage. In that case, triggering pyroptotic macrophage lysis could be a “cost” to the host that is outweighed by the other benefits of inflammasome activation.

Future studies may identify additional mechanisms of host cell death that are triggered in response to *C. albicans*. Indeed, it seems likely that the cytotoxic effect of *C. albicans* on phagocytes is a function of multiple mechanisms of cell death with factors such as phagocyte type, local environment, organism burden, and host cell activation influencing which pathway(s) is most strongly activated. Although much remains to be learned about *C. albicans*–triggered phagocyte lysis, the finding that macrophages are catching fire in response to *C. albicans* represents a paradigm shift in our understanding of *C. albicans*–phagocyte interactions. As therapies that modulate specific components of the immune response continue to be developed, a fuller understanding of programmed cell pathways in response to *C. albicans* may allow us to develop more effective treatment for this life-threatening pathogen.
